# Alloying Design Strategies for High-Performance Zn Anodes in Aqueous Zinc-Ion Batteries

**DOI:** 10.3390/ma18132997

**Published:** 2025-06-24

**Authors:** Bowen Qi, Man Huang, Ming Song, Weijia Zhou, Hua Tan

**Affiliations:** 1 Institute for Advanced Interdisciplinary Research (iAIR), School of Chemistry and Chemical Engineering, University of Jinan, Jinan 250022, China; qbw000610@163.com (B.Q.); ifc_zhouwj@ujn.edu.cn (W.Z.); 2 School of Materials and Chemical Engineering, Xuzhou University of Technology, Xuzhou 221018, China

**Keywords:** aqueous zinc-ion batteries (AZIBs), alloying strategies, zinc anodes, dendrite formation, side reactions, electrochemical stability

## Abstract

Aqueous zinc-ion batteries (AZIBs) have emerged as promising candidates for large-scale energy storage due to their inherent safety, low cost, and environmental sustainability. However, in practical applications, AZIBs are constrained by the adverse reactions originating from the zinc anodes, including dendrite formation, hydrogen evolution reaction, corrosion, and passivation, which hinder their large-scale commercialization. Nowadays, alloying strategies have been recognized as efficient approaches to address these limitations and have gained significant attention. By introducing heterogeneous elements into Zn matrices, alloying strategies can suppress dendrite formation and side reactions, modulate the interfacial kinetic process, and enhance electrochemical stability. This review systematically discusses the advantages of alloying for Zn anodes, categorizes key design strategies, such as surface modifications, composite structures, functional alloying, gradient, and layered alloy designs, and meanwhile highlights their performance improvements. Furthermore, we suggest future directions for advanced alloy development, scalable fabrication design, and integrated system optimization. Alloy engineering represents a critical pathway toward high-performance, durable Zn anodes for next-generation AZIBs and other metal-ion batteries.

## 1. Introduction

Nowadays, aqueous zinc-ion batteries (AZIBs) have attracted substantial interest and are regarded as one of the most promising next-generation energy storage systems owing to their intrinsic safety, cost-effectiveness, and environmental friendliness compared to conventional lithium-ion batteries [1,2,3]. Zinc-ion batteries (ZIBs) structurally mirror lithium-ion batteries, as they comprise a cathode, electrolyte, separator, and anode. The cathode, coated on a current collector, integrates an active material, a conductive additive, and a binder; prominent active materials include manganese-based compounds (e.g., MnO_2_) [4,5], vanadium-based compounds (e.g., VO_x_) [6,7], conductive polymers [8,9], and Prussian blue analogues (PBAs) [10,11,12], selected for their superior electrochemical performance. The electrolyte, crucial for ion transport and determining the voltage window and conductivity, predominantly utilizes aqueous systems (neutral/mildly acidic) with salts like ZnSO_4_ [13], ZnCl_2_ [14,15], Zn(CF_3_SO_3_)_2_ [16,17,18], or Zn(TFSI)_2_ [19,20]. The separator requires electronic insulation and ionic conductivity; traditional glass fiber variants [21,22] form porous structures via random stacking of borosilicate fibers, with inherent hydrophilicity ensuring effective electrolyte wetting and enhancing cycling stability. The dominant anode materials are metallic zinc foil [23,24] and zinc powder [25,26,27], favored for their excellent conductivity and highly reversible zinc deposition/stripping behavior.

**Figure 1:**
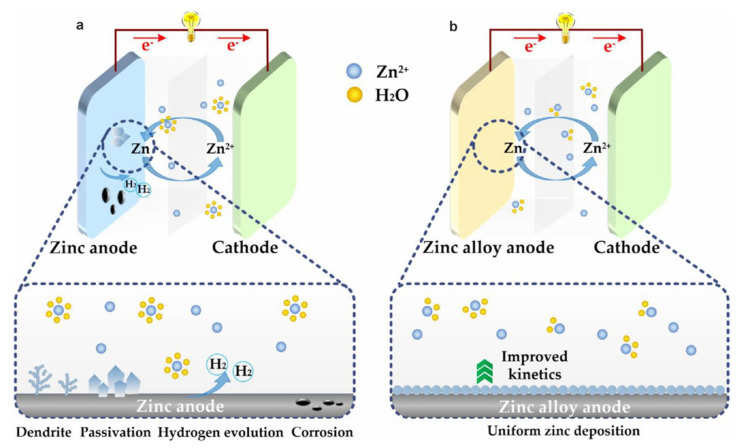
Schematic illustration of interfacial zinc deposition behaviors on (**a**) zinc anode and (**b**) zinc alloy anode. Ref. [58] Copyright 2023, American Chemical Society.

In AZIBs, this eliminates flammability risks due to the use of aqueous electrolytes, while the natural abundance and high theoretical capacity (820 mAh g^−1^) of zinc (Zn) make AZIBs particularly attractive for grid-scale energy storage and portable electronics [28,29,30]. In real applications, despite these advantages, the practical deployment of AZIBs is hindered by critical challenges associated with Zn anodes, including uncontrolled dendrite growth, hydrogen evolution reaction (HER), corrosion, and passivation [31,32,33,34]. Zinc dendrites arise from uneven Zn^2+^ migration and tip-induced anisotropic deposition, leading to active material loss, reduced Coulombic efficiency, and potential internal short circuits [35,36,37]. Concurrently, HER and corrosion, as competing interfacial side reactions, also degrade battery performance, consume electrons and water, and produce hydrogen bubbles that block ion transport and cause local pH fluctuations, inducing the formation of insulating byproducts and destabilizing the electrode interface [38,39,40]. Moreover, corrosion continuously consumes zinc, even at rest, generating gas and products, increasing interfacial resistance and compromising mechanical integrity [23,41,42]. These processes collectively accelerate active material loss and electrolyte depletion, resulting in rapid capacity fading. Therefore, these issues lead to poor cycling stability, low coulombic efficiency, and even premature battery failure, significantly impeding the commercialization of AZIBs (Figure 1a) [43,44,45].

To overcome the limitations mentioned above, various strategies have been explored, such as electrolyte optimization [46,47,48], interface engineering [49,50,51], and structural design of Zn anodes [52,53,54]. Among these approaches, alloying strategies have emerged as a highly effective method for enhancing the electrochemical performance of Zn anodes [55,56,57] by introducing heterogeneous elements into Zn matrices. Moreover, alloying strategies can effectively suppress dendrite growth and side reactions, achieve homogeneous distribution of active sites, promote interfacial kinetics, and enhance mechanical/electrochemical stability, thereby extending the lifespan of AZIBs and demonstrating significant application prospects [1,58,59] (Figure 1b).

In this review, we systematically summarize the recent progress in alloying strategies for high-performance zinc anodes, delving into the intrinsic mechanisms by which alloying suppresses dendrite formation, inhibits side reactions, and optimizes reaction kinetics. We focus on elucidating the following key design principles: homogeneous alloys, surface modification, functionalized alloys, composite structures, and gradient/layered architectures. Furthermore, we provide in-depth perspectives on the challenges and future prospects of alloy engineering, proposing that future core research on alloyed zinc matrices should focus on three key aspects: multifunctional material design, scalable fabrication techniques, and system integration optimization. Finally, the systematically outlined alloying modulation strategies for zinc anodes presented herein provide important theoretical guidance and design insights for developing the next generation of highly efficient and durable AZIBs and anodes for other metal-ion batteries.

## 2. Advantages of Alloying Strategies for Zn Anodes

### 2.1. Dendrite Suppression via Alloying

In aqueous zinc-ion batteries (AZIBs), the uncontrolled growth of Zn dendrites significantly compromises cycling stability and operational safety, making resolving this critical issue paramount. The alloying strategy has been demonstrated as an effective approach to regulate Zn deposition behavior [60,61,62]. Introducing alloying elements (e.g., Cu) can alter the electronic structure of the Zn matrix and reduce surface energy heterogeneity, thereby constructing favorable nucleation sites [63,64,65]. For instance, Tian et al. [63] constructed a three-dimensional Zn-Cu alloy anode material, which promoted uniform Zn nucleation by enhancing the binding energy to Zn and lowering the nucleation barrier. Similarly, in the Zn-Cu alloy prepared by Zhou et al. [64], Cu sites exhibited stronger adsorption capability toward Zn^2+^, inducing uniform Zn deposition on the electrode surface. Zhang et al. [65] also demonstrated that Zn-Cu alloys facilitate uniform Zn deposition and suppress dendrite formation by enhancing Zn affinity and reducing nucleation overpotential. This atomic-level modification strategy promotes uniform adsorption and subsequent deposition of Zn ions, effectively suppressing preferential growth at protrusions on the electrode surface.

From an electrochemical perspective, alloy phases possess unique work function characteristics capable of redistributing interfacial charge density, thereby homogenizing the local electric field and current distribution [66,67,68]. This effect mitigates concentration polarization, which drives dendrite growth. For example, the Zn-Cu alloy prepared by Liu et al. [66] exhibited a compositional gradient from CuZn_5_ at the bottom to Cu_5_Zn_8_ at the top; differences in the electronic structure of distinct phases led to a gradient in work function, driving charge redistribution and forming a uniform potential field. Du et al. [67] prepared a Zn-Bi alloy, where Bi incorporation modified the alloy’s electronic structure; the high proportion of Zn(002) crystal planes synergized with Bi-induced lattice distortion to optimize work function distribution, promoting uniform electric field distribution and inhibiting dendrite formation. Furthermore, Wu et al. [68] successfully fabricated a Zn-In alloy phase; the addition of In optimized the electronic structure, and its synergy with a three-dimensional carbon network further enabled uniform distribution of the local electric field and current. Moreover, the alloying strategy significantly influences the electrical double layer (EDL) structure at the electrode-electrolyte interface, thereby altering Zn^2+^ diffusion kinetics and deposition overpotential. By modulating interface energetics, interfacial modification selectively promotes lateral Zn deposition while suppressing its vertical growth mode. This mechanism fundamentally inhibits dendrite formation, effectively enhancing the stability and safety of the electrode system. The Zn-Cu alloy prepared by Meng et al. [56] enabled precise regulation of Zn deposition behavior by modifying ion adsorption behavior, potential distribution, and interfacial impedance within the EDL. The nanoscale interface in the Zn-Ag alloy developed by Chen et al. [69] increased the effective surface area of the EDL, promoting uniform charge distribution and effectively achieving uniform and reversible interfacial deposition. The Zn-Al alloy constructed by Wang et al., utilizing a layered nanostructure and core-shell interface, reconfigured the charge distribution and ion migration pathways within the EDL [70].

In the field of metal-ion batteries, the alloying strategy is widely employed to regulate the microstructure of metal anodes. For instance, in lithium-ion batteries, introducing Al_2_Yb to form Li-3Al-1Yb alloy induces grain refinement through a triple mechanism of heterogeneous nucleation (increasing nucleation sites), high adsorption energy (regulating Li^+^ deposition behavior), and grain boundary activity (optimizing electrochemical reactions), effectively suppressing dendrite growth [71]. In sodium-ion batteries, a Sn-Bi-Sb ternary alloying strategy utilizes solid solution formation, lattice distortion, and composition regulation to achieve grain refinement [72]. In magnesium-ion batteries, the nanostructured design of Bi-Sn alloy significantly refines grain size and enhances electrode structural stability through phase boundary/grain boundary synergistic effects [73]. These processes exemplify the common mechanism of alloying elements in regulating the microstructure of metal anodes. However, compared to other metal anodes, the alloying regulation of the Zn anode exhibits distinct uniqueness. Although both Zn and Mg possess a hexagonal close-packed (hcp) structure, Zn exhibits greater deviation from the ideal hcp configuration and stronger anisotropy. This leads to a greater tendency for non-uniform ion flux and dendrite growth during deposition, phenomena largely absent in lithium/sodium anodes with body-centered cubic (bcc) structures. By inducing grain refinement and texture evolution via alloying, the Zn anode can form a more isotropic matrix structure, homogenizing Zn^2+^ flux, promoting uniform Zn nucleation, and ultimately enhancing deposition uniformity while suppressing dendrite formation. Additionally, the presence of alloy phases alters the mechanical properties of the deposition substrate, enhancing its resistance to deformation under stresses induced by repeated Zn deposition-stripping cycles (e.g., Zn-Ce [74], Zn-Cu [75], and Zn-Zr [76]). By integrating regulatory mechanisms across atomic, electrochemical, and microstructural scales, these intertwined effects synergistically confer alloyed Zn anodes with superior dendrite suppression capability, establishing alloy engineering as a core strategy for achieving stable Zn deposition in advanced aqueous zinc-ion battery systems.

### 2.2. Suppression of HER Parasitic Reactions via Alloying Strategy

In addition to the advantages mentioned above, alloying strategies can also effectively address the key challenges in zinc anodes by suppressing parasitic hydrogen evolution (HER) and corrosion reactions [77,78,79]. These detrimental processes, inherent to zinc’s high reactivity in aqueous electrolytes, degrade battery performance through active material consumption, electrolyte depletion, and interface deterioration, leading to reduced coulombic efficiency and accelerated capacity fading. Accordingly, alloying by incorporating high-HER-overpotential metals (In, Sb, Sn, Cr, and Hg) [70,80,81,82,83,84] is an effective strategy to address the above-mentioned issues. The approach works by simultaneously raising the thermodynamic barrier for HER initiation while kinetically hindering proton adsorption [85,86,87,88]. This synergistic effect stabilizes the electrochemical interface through electronic structure modification and enhanced corrosion resistance.

Remarkably, alloy engineering addresses the fundamental causes rather than merely alleviating symptoms of anode degradation. The cumulative impact of these alloying effects manifests as significantly improved interfacial stability during extended cycling operations (e.g., Cu-Sn [89], Zn-Fe-Ni [90], and In-Sn [91]). This strategic approach not only enhances immediate electrochemical performance but also contributes to the development of more robust and durable energy storage systems, paving the way for practical implementation of aqueous zinc-based battery technologies in demanding applications [92,93,94]. The continued refinement of alloy compositions and the exploration of novel alloying elements promise further advancements in the pursuit of ultimately stable zinc anode systems.

### 2.3. Kinetics Optimization Through Alloy Engineering

Alloy engineering induces profound modifications in the electrochemical kinetics of zinc deposition and dissolution processes in aqueous battery systems (e.g., Zn-Cu [95], Zn-P [96], and Zn-Al [97]). The incorporation of foreign metal atoms fundamentally restructures the electronic environment of the zinc matrix, generating localized charge polarization effects that substantially reduce the activation energy barriers for electrochemical transformations. This electronic restructuring manifests most prominently at the electrode–electrolyte interface, where alloy components with carefully tuned work functions establish highly efficient charge transfer pathways during redox cycling [98,99,100].

Meanwhile, the adjusted binding energetics between zinc ions and the alloy surface collectively optimize the interplay between ion adsorption thermodynamics and surface diffusion kinetics [101,102]. Consequently, alloying can enhance the nucleation dynamics of zinc deposition through the creation of a modified surface energy, providing optimized coordination environments for zinc adatom incorporation (e.g., Zn-Ag and Zn-Cu [103]).

At the electrolyte interface, alloy-modified surfaces exert significant influence on Zn^2+^ solvation dynamics through precisely tailored interfacial potential gradients. The restructured electrical double layer facilitates the partial desolvation of hydrated zinc complexes, substantially reducing the energetic penalty associated with this typically rate-limiting process (e.g., Zn-Sn-Bi [104], Zn-Ti [105], and Zn-Ag [106]). Concurrently, the engineered alloy surface chemistry modulates water molecule interactions, effectively suppressing the competing hydrogen adsorption processes that would otherwise interfere with zinc deposition kinetics.

These coordinated modifications yield substantial improvements in reaction rates for both zinc plating and stripping processes. Thus, the optimized kinetics promote uniform current distribution across the electrode surface, effectively eliminating localized current concentration effects that frequently lead to irregular deposition patterns, representing a holistic solution to the intrinsic limitations of zinc anode systems in aqueous electrolytes (e.g., Zn-Cu [107,108] and Zn-Sn-Pb [109]). The investigations into kinetic enhancement mechanisms will inform the development of advanced zinc alloy formulations with superior power characteristics and cycling stability.

## 3. Fundamental Alloying Strategies for Zinc Anodes

**Figure 2:**
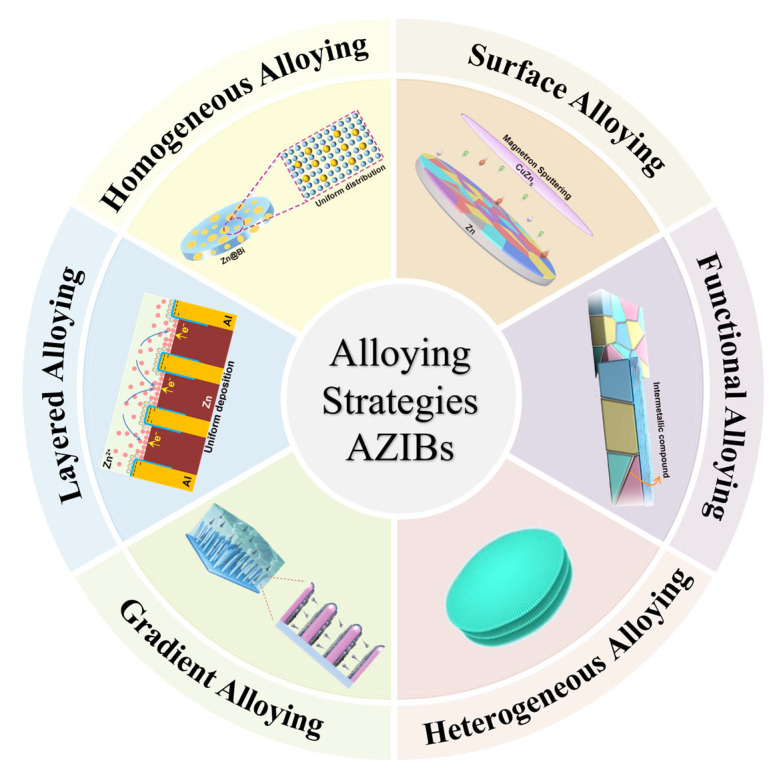
Key alloy design strategies for zinc anodes in AZIBs [59,67,70,105,110,111].

As mentioned above, alloying strategies represent effective approaches for regulating zinc deposition behavior and mitigating heterogeneous dendrite formation and other side reactions in AZIBs to solve the challenges from anode-related degradation processes, which can precisely tune the anode performance through intrinsic material property optimization. In this section, we will comprehensively examine the principal alloying strategies as follows (as shown in Figure 2): (i) bulk-phase homogeneous alloying, (ii) surface alloy engineering strategies, (iii) functional alloying strategies, (iv) heterogeneous composite alloying, (v) gradient alloying designs, and (vi) layered alloying designs—each offering distinct advantages for zinc anode stabilization.

### 3.1. Bulk-Phase Homogeneous Alloying in Zinc Anodes

**Figure 3:**
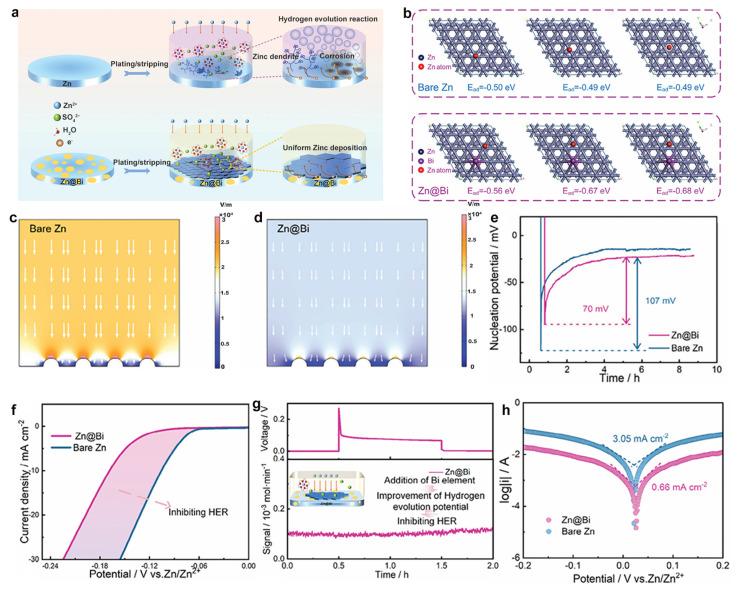
(**a**) Zn deposition mechanisms on bare Zn and Zn-Bi alloy. (**b**) DFT-optimized Zn^2+^ adsorption configurations on bare Zn and Zn-Bi surfaces. (**c**) Nucleation overpotentials in Zn//Cu and Zn-Bi//Cu cells. Electric field distributions of (**d**) bare Zn and (**e**) Zn-Bi. (**f**) HER activity comparison in 1 M Na_2_SO_4_. (**g**) In situ DEMS spectrum of Zn-Bi symmetric cells. (**h**) Corrosion resistance evaluation of bare Zn and Zn–Bi. Ref. [67] Copyright 2023, Wiley-VCH.

Homogeneous alloy systems, characterized by atomic-level uniform distribution of constituent elements, offer an effective approach to modulate the intrinsic physicochemical properties of Zn anodes. Du et al. [67] demonstrated this through the successful fabrication of a Zn–Bi homogeneous alloy anode using a high-temperature melting method. Comprehensive microstructural characterization confirmed the homogeneous dispersion of Bi atoms throughout the zinc matrix (Figure 3a). Density functional theory (DFT) calculations revealed that Bi incorporation substantially enhances Zn atom adsorption energy, leading to reduced nucleation overpotential and accelerated deposition kinetics (Figure 3b–c). The Bi component simultaneously homogenizes the surface electric field distribution, mitigating localized charge accumulation while providing uniform nucleation sites for Zn^2+^ ions. This dual effect promotes homogeneous Zn deposition and effectively suppresses dendrite formation (Figure 3d–e).

In addition, the electrochemical characterization further demonstrated the advantages of this alloy system. The Bi component exhibits remarkably low hydrogen evolution reaction (HER) activity, with linear sweep voltammetry (LSV) confirming a significantly reduced HER overpotential. Differential electrochemical mass spectrometry (DEMS) measurements provided additional evidence of minimal H_2_ generation (Figure 3f–g). Furthermore, the corrosion-resistant nature of Bi substantially improves the alloy’s electrochemical stability, yielding a lower corrosion current density (0.62 vs. 1.45 mA cm^−2^) and higher corrosion potential (−0.97 vs. −1.03 V) compared to pure Zn, thereby effectively inhibiting corrosion-related side reactions (Figure 3h). These collective improvements highlight the potential of homogeneous alloying as a robust strategy for stabilizing Zn anodes in practical applications.

### 3.2. Surface Alloy Engineering Strategies for Zinc Anodes

**Figure 4:**
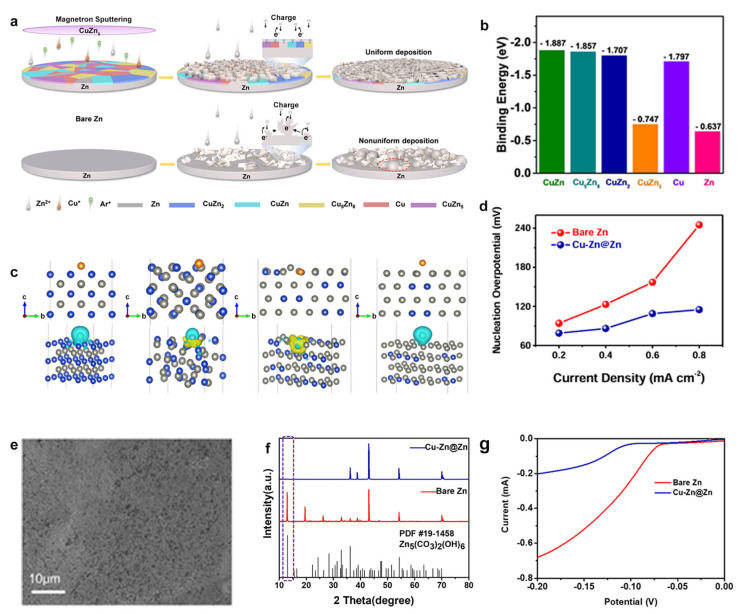
(**a**) Fabrication process of Cu–Zn@Zn electrode and its Zn deposition behavior. (**b**) DFT-calculated Zn adsorption energies on various Cu–Zn alloys. (**c**) Atomic configurations and charge density distributions of Zn adsorption on different Cu–Zn phases. (**d**) Comparison of nucleation overpotentials at different current densities. (**e**) Surface morphology of the cycled Cu–Zn@Zn electrode. (**f**) Phase analysis of X-ray diffraction (XRD) patterns after 40 cycles at 1 mA cm^−2^. (**g**) HER polarization curves in 0.01 M HCl electrolyte. Ref. [59] Copyright 2022, Wiley-VCH.

Surface alloying is one of the most effective modification strategies, which creates a protective alloy layer on zinc substrates through physical or chemical deposition techniques, substantially improving anode electrochemical performance. This approach offers dual benefits: the alloy layer serves as a physical barrier that minimizes direct zinc–electrolyte contact to suppress parasitic reactions, while simultaneously providing favorable nucleation sites for homogeneous Zn^2+^ deposition. Li et al. [59] developed a high-performance Cu–Zn surface alloy through magnetron sputtering deposition (Figure 4a). DFT calculations revealed the Cu–Zn alloy layer enhanced the adsorption capability of the Zn atom compared to pure zinc, lowering nucleation overpotential by approximately 35% and providing faster ion transport kinetics (Figure 4b–d).

Furthermore, the scanning electron microscope (SEM) analysis showed that the engineered interface exhibited remarkable stability with the Cu–Zn@Zn electrode after 100 cycles owing to the smooth, dendrite-free surface and showed nondetectable byproducts even after 40 cycles (Figure 4e–f). The LSV measurements, which showed a 120 mV increase in hydrogen evolution overpotential compared to bare zinc (Figure 4g), demonstrated the alloy layer’s effectiveness in suppressing side reactions. These results systematically demonstrate that surface alloying would simultaneously optimize the deposition kinetics, physical isolation from corrosive electrolytes, and thermodynamic suppression of parasitic reactions in zinc anodes. The surface alloy engineering presents that it is a viable pathway for developing durable zinc anodes capable of withstanding extended cycling in aqueous battery systems.

### 3.3. Functional Alloying Strategies for Zinc Anodes

**Figure 5:**
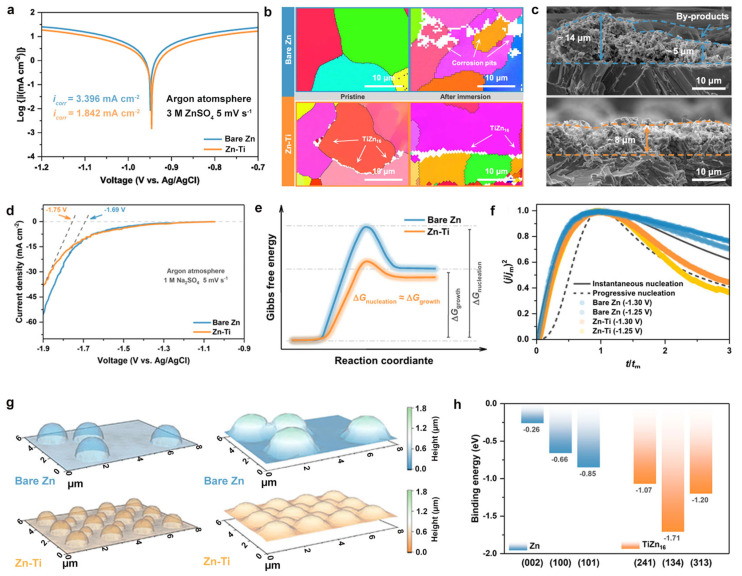
(**a**) Tafel plots. (**b**) EBSD IPF maps of Zn anodes (pristine vs. 24 h immersion). Color codes: crystal orientations; White regions: corrosion pits/TiZn_16_ IMCs. (**c**) Cross-sectional SEM images after 50 cycles. (**d**) LSV curves recorded under argon atmosphere. (**e**) Gibbs free energy evolution during Zn deposition. (**f**) Experimental vs. theoretical 3D nucleation transients. (**g**) Simulated Zn deposition: initial nuclei vs. 240 s plated state. (**h**) Calculated binding energies of Zn atom with different crystal facets. Ref. [105] Copyright 2023, Springer Nature.

Functional alloying has emerged as a pivotal strategy to tackle the inherent challenges of zinc anodes in AZIBs, such as the interfacial instability and dendrite formation. The alloy-based designs enable precise modulation of zinc deposition thermodynamics and kinetics by synergistically tailoring composition and microstructure. Zhao et al. [105] fabricated a Zn-based dual-phase alloy through metallurgical methods, where TiZn_16_ intermetallic compounds preferentially segregate at the grain boundaries. This microstructure can effectively passivate reactive boundaries, suppressing intergranular corrosion and enhancing interfacial stability. Electrochemical measurements reveal that the alloy exhibits a higher corrosion potential and lower corrosion current density (1.915 vs. 3.497 mA cm^−2^), indicating a significantly mitigated corrosion tendency (Figure 5a). EBSD analysis shows that after 24 h of immersion, bare Zn suffers severe grain boundary corrosion, while the Zn–Ti alloy retains the Zn structure with TiZn_16_ phases remaining clearly visible (Figure 5b). Cross-sectional SEM images confirm a compact, byproduct-free deposition layer on the alloy surface, contrasting with porous pure Zn (Figure 5c). The alloy exhibits suppressed hydrogen evolution activity, which can be confirmed by the reduced LSV currents under Ar atmosphere (Figure 5d).

Nucleation kinetics analysis identifies the hybrid mechanism of merging instantaneous and progressive modes (Figure 5e), driven by reduced energy barriers (ΔG_nucleation_ − ΔG_growth_) that accelerate charge transfer (Figure 5f). The nucleation Gibbs free energy (ΔG_nucleation_) represents the energy barrier required to form a critical-sized nucleus as comprising two components: the volumetric free energy (ΔG_v_) and the surface free energy (ΔG_s_). ΔG_v_ corresponds to the reduction in the system’s free energy during nucleus formation, while ΔG_s_ represents the energy consumption required to create the new phase interface. The growth Gibbs free energy (ΔG_growth_) refers to the energy required for the subsequent growth of the nucleus [112]. When ΔG_nucleation_ is significantly higher than ΔGgrowth, nucleation is suppressed and growth dominates. This thermodynamic imbalance promotes preferential zinc ion deposition at existing nucleation sites, potentially leading to dendrite formation with increasing deposition capacity. Conversely, when ΔG_nucleation_ is reduced to a level comparable to ΔG_growth_, the homogeneous formation of more critical nuclei is significantly promoted, enabling dense and uniform zinc deposition [113]. This mechanism holds important theoretical significance for understanding morphological evolution in metal electrodeposition processes. COMSOL Multiphysics 5.6 (COMSOL) simulations corroborate uniform zinc nuclei distribution evolving into dense plating layers (Figure 5g), while DFT calculations quantify stronger zinc adsorption energy on alloy surfaces, promoting site-selective nucleation (Figure 5h). Collectively, functional alloying establishes a dual-regulation strategy—thermodynamic stabilization through IMC passivation and kinetic enhancement via nucleation facilitation—enabling uniform zinc deposition and robust interfacial stability for aqueous zinc-ion batteries.

### 3.4. Heterogeneous Composite Alloying for Zinc Anodes

**Figure 6:**
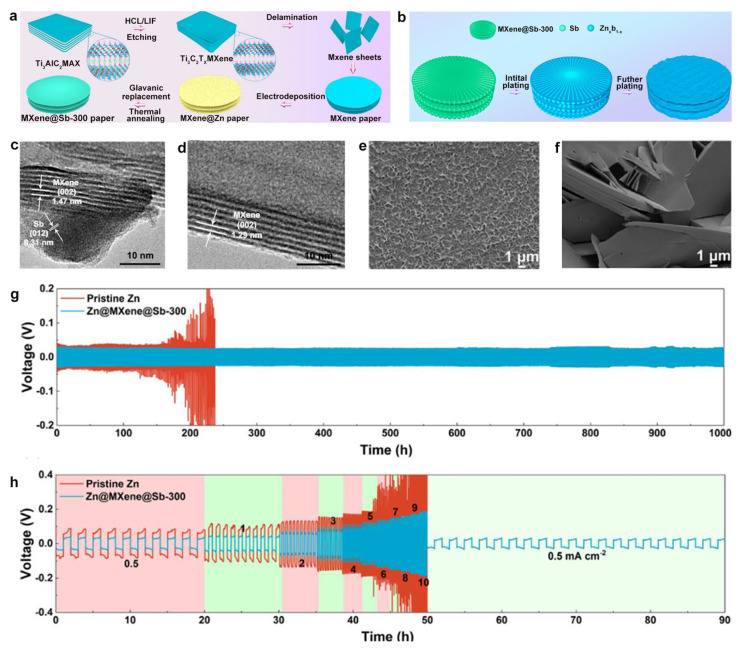
(**a**) Fabrication process schematic of MXene@Sb-300 composite. (**b**) Zinc deposition mechanism on MXene@Sb-300. (**c**,**d**) High-resolution TEM images of MXene@Sb-300 and pristine Ti_3_C_2_T_x_ MXene. (**e**,**f**) Surface morphology comparison after 100 cycles at 5 mA cm^−2^. (**g**) Galvanostatic cycling profiles at 0.5 mA cm^−2^. (**h**) Rate capability evaluation from 0.5–10 mA cm^−2^. Ref. [110] Copyright 2021, Elsevier.

Heterogeneous composite alloying represents an innovative approach to zinc anode engineering, where the strategic integration of metallic zinc with functional conductive matrices creates composite systems capable of precisely regulating deposition behavior while enhancing interfacial stability and electrochemical performance. Tian et al. [110] developed a prime example MXene@Sb-300 composite through a sophisticated combination of electrodeposition, displacement reactions, and thermal annealing at 300 °C (Figure 6a). This design capitalizes on interface engineering to strengthen the bonding between the MXene substrate and Sb nanostructure array, while enabling reversible ZnSb alloy formation during cycling that serves as efficient zinc nucleation sites, significantly reducing overpotential and promoting uniform deposition (Figure 6b).

In this composite system, the three-dimensional MXene network plays a multifaceted role, combining exceptional electronic conductivity with enhanced ionic transport through precisely tuned (002) interlayer spacing expansion from 1.29 nm to 1.47 nm (Figure 6c,d). This unique architecture simultaneously homogenizes the electrode surface electric field while providing optimized charge and mass transport pathways. This system reveals remarkable stability in stark contrast to the severe degradation observed in conventional titanium foil electrodes (Figure 6e,f). According to the electrochemical evaluation, the MXene@Sb-300 electrode demonstrated high cycling stability, exceeding 1000 h at 1 mA cm^−2^ coupled with exceptional rate capability (Figure 6g,h), establishing this heterogeneous alloying approach as a promising strategy for developing durable, high-performance zinc anodes.

### 3.5. Gradient Alloying Designs in Zinc Anodes

**Figure 7:**
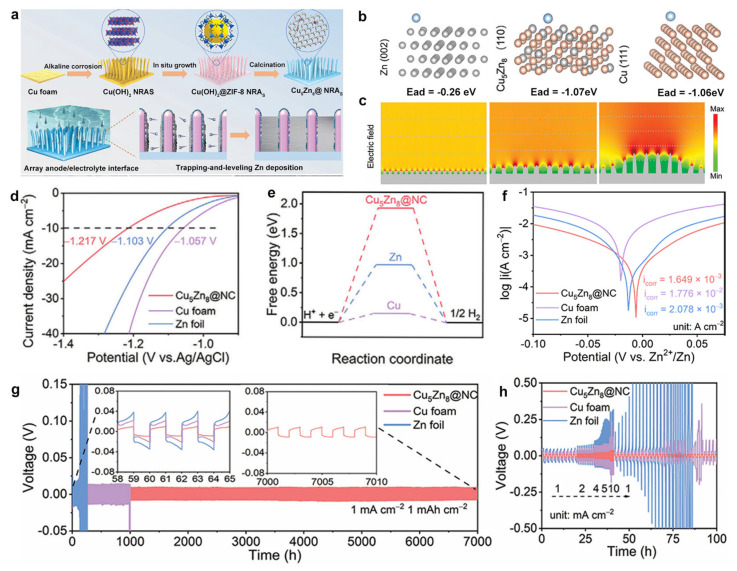
(**a**) Synthesis schematic of Cu_5_Zn_8_@NC nanorod arrays. (**b**) DFT-calculated adsorption energies and charge density distributions for Zn deposition on different crystal planes. (**c**) Simulated Zn^2+^ flux distributions during plating. (**d**) HER polarization curves at 10 mV s^−1^. (**e**) Calculated HER Gibbs free energy diagrams. (**f**) Tafel plots for corrosion evaluation. (**g**) Long-term cycling stability of symmetric cells. (**h**) Rate capability assessment. Ref. [111] Copyright 2024, Wiley-VCH.

Gradient alloy architectures have been confirmed as an advanced design strategy, creating spatially controlled compositional variations within zinc anodes and enabling synergistic integration of multiple functional components. Tian et al. [111] demonstrated this concept through the fabrication of Cu_5_Zn_8_@NC core-shell structures using an innovative alkali etching-growth approach combined with Zeolitic Imidazolate Framework-8 (ZIF-8) coating and carbonization (Figure 7a). This hierarchical design features the Cu_5_Zn_8_ alloy core with superior zinc adsorption energy, as confirmed by DFT calculations (Figure 7b). This improvement can be attributed to the abundant nucleation sites, which significantly reduce the deposition overpotential. Meanwhile, the three-dimensional porous nanorod array architecture spatially confines zinc deposition within inter-rod gaps, effectively preventing dendrite formation.

In this system, the effectiveness stems from a unique “trapping–leveling” deposition mechanism, where zinc ions first nucleate at the highly active Cu_5_Zn_8_ sites before undergoing constrained growth within the nanorod framework, owing to the uniform electric field distribution evidenced by COMSOL simulations (Figure 7c). According to the LSV and DFT-calculated Gibbs free energy analysis (Figure 7d–e), the nitrogen-doped carbon shell can physically shield the electrode contact with the electrolyte, thermodynamically suppressing hydrogen evolution. This comprehensive protection strategy demonstrated that the electrode maintained exceptional performance over 7000 h of cycling at 1 mA cm^−2^ (Figure 7g), coupled with outstanding corrosion resistance (Figure 7f) and excellent rate capability (Figure 7h). The gradient approach thus establishes a new paradigm for zinc anode design, simultaneously addressing nucleation, deposition, and interfacial stability challenges through precisely engineered material architectures.

### 3.6. Layered Alloying Designs in Zinc Anodes

**Figure 8:**
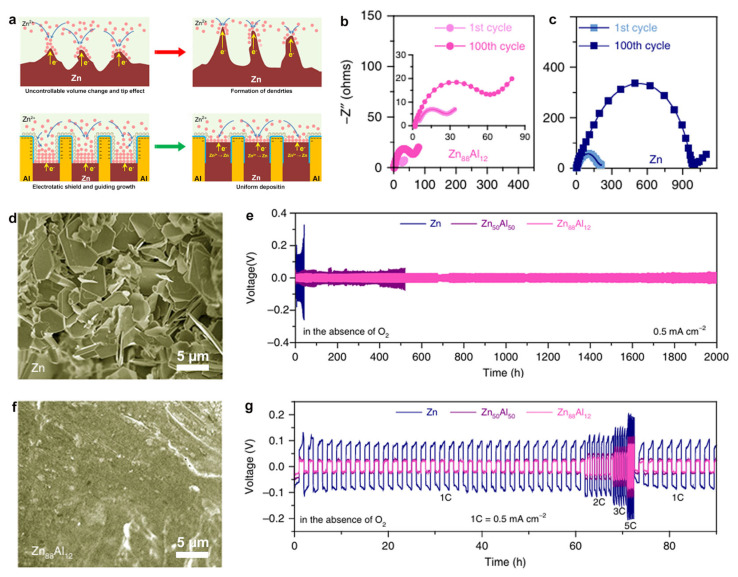
(**a**) Schematic of dendrite/crack suppression via eutectic design in Zn_88_Al_12_ vs. bare Zn anodes. EIS spectra of (**b**) eutectic Zn_88_Al_12_ (λ ≈ 450 nm) and (**c**) monometallic Zn in O_2_-free ZnSO_4_ after 1st/100th cycles. SEM images of (**d**) monometallic Zn and (**e**) eutectic Zn_88_Al_12_ after 2000 cycles and 42 h plating/stripping in O_2_-free ZnSO_4_ (Scale bar: 5 μm). (**f**) Long-term cycling stability at 0.5 mA cm^−2^. (**g**) Voltage profiles at various C-rates (1 C = 0.5 mA cm^−2^) for monometallic Zn, hypoeutectic Zn_50_Al_50_, and eutectic Zn_88_Al_12_ symmetric cells. Ref. [70] Copyright 2020, Springer Nature.

Layered alloy design can effectively address the intrinsic limitations of materials through structural stratification and interfacial synergy, featuring alternately stacked metallic phases with complementary functionalities. Wang et al. [70] fabricated a nanolayered structure with alternating zinc (hcp) and aluminum (face-centered cubic, fcc)) phases via a one-step smelting method. During cycling, the aluminum layers undergo in situ oxidation to form Al/Al_2_O_3_ core-shell structures. The insulating Al_2_O_3_ shell effectively prevents zinc ion reduction on the aluminum surface, while the electrostatic shielding effect guides uniform zinc deposition onto adjacent zinc layers, thereby suppressing dendrite growth. In contrast, bare zinc anodes exhibit severe dendritic growth under identical conditions (Figure 8a).

Meanwhile, the electrochemical characterization confirms the alloy’s superior stability: the eutectic Zn–Al alloy shows a small charge transfer resistance (RCT) change of 24 Ω after 100 cycles and maintains a smooth surface after 2000 cycles, indicating excellent stability during zinc deposition/stripping (Figure 8b,c,e). The layered design enables symmetric cells to achieve ultra-stable cycling over 2000 h at 0.5 mA cm^−2^, coupled with remarkable rate capability across current densities (Figure 8f,g). Cross-sectional analysis reveals a compact, dendrite-free morphology in the alloy, contrasting with the porous structure and byproduct accumulation observed in conventional Zn (Figure 8d). Therefore, this strategy synergizes chemical passivation and electric field modulation, establishing a dual-regulation mechanism that simultaneously enhances interfacial stability, suppresses side reactions, and accelerates ion transport kinetics. The nanolayered architecture provides a scalable paradigm for developing high-performance zinc anodes in AZIBs.

## 4. Current Challenges and Future Perspectives

Despite the remarkable progress in alloying strategies for zinc anodes, significant challenges remain in their practical implementation in AZIBs. For next-generation materials, research should extend beyond electrochemical performance to incorporate multifunctional designs featuring self-healing capabilities and enhanced mechanical properties. The development pathway must prioritize cost-effective synthesis methods and earth-abundant elements to enable scalable production, breaking through the current limits of reliance on precious metals and complex deposition techniques. Most crucially, a system-level perspective is required to understand and optimize the complex interactions among alloy anodes, diverse cathode materials, and electrolyte formulations in practical full-cell configurations. Emerging computational approaches, particularly AI-driven materials discovery and multiscale modeling, offer powerful tools to accelerate progress across all these fronts. By simultaneously addressing materials innovation, manufacturing scalability, and system integration, researchers can unlock the full potential of alloyed zinc anodes for next-generation energy storage applications.

## 5. Conclusions

In this review, we have systematically demonstrated the alloying strategies for modulating zinc anodes in AZIBs, such as inhibiting dendrite growth, suppressing HER, interfacial corrosion, and electrode passivation. The strategic incorporation of metallic or non-metallic elements into zinc matrices enables precise control over phase composition and microstructure, leading to optimized zinc deposition morphology, stabilized interfaces, and enhanced reaction kinetics. This review comprehensively summarizes six key alloying approaches—homogeneous bulk alloys, surface alloys, functional alloying, heterogeneous composites, gradient, and layered architectures—highlighting their respective mechanisms and performance advantages. While these innovations show great promise in laboratory settings, in practice, alloying strategies still face substantial barriers related to scalable manufacturing costs and system-level compatibility. Therefore, we still need to consider and promote the development of cost-effective synthesis methods using earth-abundant elements while simultaneously optimizing electrolyte formulations and cathode materials. Currently, the integration of advanced computational tools, particularly AI-driven materials discovery and multiscale modeling, with traditional experimental approaches presents an exciting opportunity to accelerate the development of high-performance alloyed zinc anodes. Such interdisciplinary efforts will be crucial for addressing the remaining challenges and realizing the full potential of AZIBs in next-generation energy storage applications.

## Data Availability

No new data were created or analyzed in this study. Data sharing is not applicable to this article.

## Abbreviations

The following abbreviations are used in this manuscript:

AZIBs	Aqueous zinc-ion batteries
Zn	Zinc
HER	Hydrogen evolution reaction
DEMS	Differential electrochemical mass spectrometry
LSV	Linear sweep voltammetry
DFT	Density functional theory
COMSOL	COMSOL Multiphysics
SEM	Scanning electron microscope
XRD	Phase analysis of X-ray diffraction
ZIF-8	Zeolitic Imidazolate Framework-8
hcp	Hexagonal Close-Packed
fcc	Face-Centered Cubic
bcc	Body-Centered Cubic
